# Structured chaos shapes spike-response noise entropy in balanced neural networks

**DOI:** 10.3389/fncom.2014.00123

**Published:** 2014-10-02

**Authors:** Guillaume Lajoie, Jean-Philippe Thivierge, Eric Shea-Brown

**Affiliations:** ^1^Nonlinear Dynamics Department, Max Planck Institute for Dynamics and Self-OrganizationGoettingen, Germany; ^2^Bernstein Center for Computational Neuroscience, Max Planck Institute for Dynamics and Self-OrganizationGoettingen, Germany; ^3^Applied Mathematics Department, University of WashingtonSeattle, WA, USA; ^4^School of Psychology and Center for Neural Dynamics, University of OttawaOttawa, ON, Canada; ^5^Physiology and Biophysics Department, University of WashingtonSeattle, WA, USA

**Keywords:** neural variability, chaotic networks, neural excitability, network dynamics, spiking stimulus responses

## Abstract

Large networks of sparsely coupled, excitatory and inhibitory cells occur throughout the brain. For many models of these networks, a striking feature is that their dynamics are chaotic and thus, are sensitive to small perturbations. How does this chaos manifest in the neural code? Specifically, how variable are the spike patterns that such a network produces in response to an input signal? To answer this, we derive a bound for a general measure of variability—spike-train entropy. This leads to important insights on the variability of multi-cell spike pattern distributions in large recurrent networks of spiking neurons responding to fluctuating inputs. The analysis is based on results from random dynamical systems theory and is complemented by detailed numerical simulations. We find that the spike pattern entropy is an order of magnitude lower than what would be extrapolated from single cells. This holds despite the fact that network coupling becomes vanishingly sparse as network size grows—a phenomenon that depends on “extensive chaos,” as previously discovered for balanced networks without stimulus drive. Moreover, we show how spike pattern entropy is controlled by temporal features of the inputs. Our findings provide insight into how neural networks may encode stimuli in the presence of inherently chaotic dynamics.

## 1. Introduction

If a time-dependent signal is presented to a network whose dynamics are chaotic and whose initial conditions cannot be perfectly controlled, how much variability can one expect in its responses? Such a scenario is central to questions of stimulus encoding in the brain.

In this article, we study population level spiking responses in a neural network model with sparse, random connectivity and *balanced* excitation and inhibition. Such models are ubiquitous in neuroscience, and reproduce the irregular firing that typifies cortical activity. Moreover their autonomous activity is known to be chaotic, with extremely strong sensitivity of spike outputs to tiny changes in a network's initial conditions (van Vreeswijk and Sompolinsky, 1998; London et al., 2010; Sun et al., 2010). Remarkably, in these autonomous systems, the chaos is invariant to the network scale (i.e., it is *extensive*): the same spectrum of Lyapunov exponents recurs regardless of network size, even when coupling remains localized (Monteforte and Wolf, 2010; Luccioli et al., 2012). Our goal is to add a stimulus drive, and understand the implications for the network spike patterns that result—a task made challenging by the fact that spikes are related to phase space dynamics in a highly non-linear way.

Intriguingly, when such chaotic networks respond to time-dependent signals, they produce spiking that is less variable than one might expect (c.f. Molgedey et al., 1992; Marre et al., 2009; Rajan et al., 2010). In recent theoretical work, this has been attributed to low-dimensional chaotic attractors that “project” only intermittently to produce variable spiking in any given single cell (Lajoie et al., 2013). It is unclear how such chaos-induced “noise” affects neural activity in the brain. However, chaotic dynamics appears to be a general attribute of many large models of recurrent networks, a phenomenon that likely constrains biological network dynamics. Furthermore, stimulus-evoked spike data similar to that of chaotic models has been experimentally observed *in vivo*, where fluctuating sensory stimuli are repeatedly presented to an animal. Here, cortical neurons produce spikes with a wide range of variability, with some spikes repeatedly evoked with millisecond precision (Reinagel and Reid, 2000; Yang et al., 2008). Information theoretic methods suggest that this type of “intermittent noise” may permit information to be encoded in the spike patterns that single neurons produce over time (Reinagel and Reid, 2000; Tiesinga et al., 2008).

However, the impact of variability on network coding cannot be understood by extrapolating from single cells alone (Zohary et al., 1994; Abbott and Dayan, 1999; Averbeck et al., 2006; Schneidman et al., 2006; Ecker et al., 2011; Hu et al., 2014). Thus, to eventually understand how network chaos impacts coding, we need to capture the *multicell* spike train variability in chaotic networks—and relate this to well-quantified measurements at the level of single cells. Direct, sampling-based approaches to this problem will fail, due to the combinatorial explosion of spike patterns that can occur in high-dimensional networks. Another method is needed.

Studies of variability in recurrent networks typically address two distinct properties. On one hand, there is the question of spike-timing variability, often measured by binarized spike pattern entropy and usually studied for single cells or small cell groups (Strong et al., 1998; Reinagel and Reid, 2000; Schneidman et al., 2006). On the other hand, recent theoretical work investigates the dynamical entropy production of entire networks, quantifying the state space expansion globally (Monteforte and Wolf, 2010; Luccioli et al., 2012). It is not clear how these two quantities are related. Here, we extend the work of Lajoie et al. (2013) to bridge this gap, leveraging random dynamical systems theory to develop a direct symbolic mapping between phase-space dynamics and binary spike pattern statistics.

The result is a new bound for the variability of joint spike pattern distributions in large spiking networks that receive fluctuating input signals. This bound is in terms of spike-response noise entropy, an information-theoretic quantity that is directly related to dynamical entropy production. By verifying that the previous extensivity results of Monteforte and Wolf (2010) and Luccioli et al. (2012) continue to hold in the presence of stimulus drive, we show how the bound applies to networks of all sizes, and only depends on input statistics and single-cell parameters.

We then apply this bound to make two observations about the spike-pattern variability in chaotic networks. The first is that the joint variability of spike responses across large networks is at least an order of magnitude lower than what would be extrapolated from measurements of spike-response entropy in single cells, despite noise correlations that are very low on average. Second, we show that the spike-response entropy of the network as a whole is strongly controlled by the tradeoff between the mean (i.e., DC) and higher-frequency components of the input signals. Entropy increases monotonically with the mean input strength by almost an order of magnitude, even as network firing rates remain constant.

## 2. Materials and methods

### 2.1. Model

**Figure 1:**
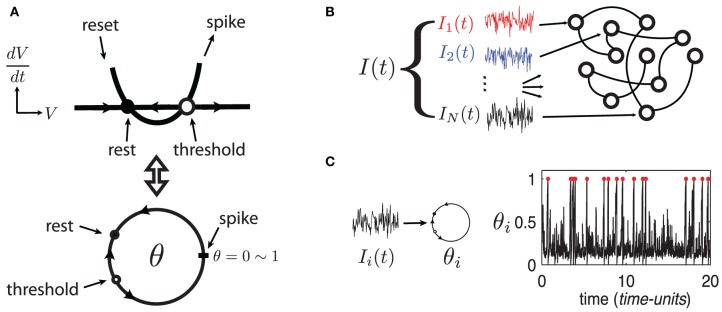
**(Color online) (A)** Sketch of equivalent dynamics between Quadratic-Integrate-and-Fire and the θ-neuron. **(B)** Cartoon representation of a network driven by a quenched collection of inputs *I*(*t*) = {*I*_*i*_(*t*)}_*i* = 1, …, *N*_. **(C)** Example of a single θ-neuron θ_*i*_ in response to a (quenched) input *I*_*i*_(*t*) (η = −0.5, ε = 0.5). Red dots mark spike times.

To develop these results, we use large random networks of *N* Quadratic Integrate-and-Fire (QIF) model neurons, as in Monteforte and Wolf (2010) and Lajoie et al. (2013). This single neuron model captures the normal form dynamics of Type I neurons, as found in cortex (Ermentrout, 1996). Moreover, we make use of a smooth change of coordinates that maps QIF hybrid dynamics to a phase variable on the unit circle (see Ermentrout, 1996 and appendix of Lajoie et al., 2013). This cell model is known as the “θ-neuron” and eliminates the need for artificial reset after a spike. This results in smooth dynamics with dimensionless units, a feature which will prove crucial for analysis (see Figure 1A). For reference, in a QIF model neuron with a time constant τ = 10 ms, one *time-unit* in the θ-coordinates corresponds to about 125 ms.

The state of each cell in the network is represented by a phase variable θ_*i*_(*t*) ∈ [0, 1] (*i* = 1, …, *N*) where 0 and 1 are identified (i.e., *S*^1^) and a spike is said to occur when θ_*i*_ = 1 ~ 0. In addition to internal dynamics which depend on coupling between neurons, the network receives a temporally structured input signal *I*(*t*), as described below.

The dynamics of the *i*th cell in the network are given by the equation

(1) $$\begin{array}{l} d\theta_{i}=[F(\theta_{i})+Z(\theta_{i})\sum_{j\;=\;1}^{N}a_{ij}g(\theta_{j})+\frac{\varepsilon^{2}}{2}Z(\theta_{i})Z^{\prime }(\theta_{i})]dt\ldots \\ \;+Z(\theta_{i})\underset{I_{i}(t)dt}{\underset{︸}{\left[\eta dt+\varepsilon dW_{i,t}\right]}} \end{array}$$

where *F*(θ_*i*_) = 1 + cos(2πθ_*i*_), *Z*(θ_*i*_) = 1 − cos(2πθ_*i*_) and

$$g(\theta_{j})=\left\{ \begin{array}{l} d{\left(b^{2}-{\left[\left(\theta_{i}+\frac{1}{2}\right)\text{mod}1-\frac{1}{2}\right]}^{2}\right)}^{3};\;\theta_{i}\in [-b,b]\\ \;0\text{ ; else} \end{array}\right.$$

is a smooth coupling function with small support around θ_*j*_ = 1 ~ 0, mimicking the rapid rise and fall of a synaptic current (*b* = 1/20, *d* = 35/32). The ε^2^ term comes from an Ito correction (Lindner et al., 2003).

The network's input *I* = {*I*_*i*_}^*N*^_*i* = 1_, represented by the last term in (1), models a temporal stimulus. It is a collection of *N* independent signals *I*_*i*_(*t*) = η + ε *dW*_*i*,*t*_/*dt* driving each neuron respectively, where the *dW*_*i*,*t*_/*dt* are quenched realizations of white noise—that is, scaled increments of the independent Wiener processes *W*_*i*,*t*_ (see Figure 1B). Note that the input's mean η controls the network's “excitability” and can take negative values (Ermentrout, 1996) while ε ≥ 0 controls the amplitude of input fluctuations. Both parameters are constant across all cells. We begin by investigating network (1) in the excitable regime with parameters η = −0.5 and ε = 0.5. Figure 1C shows an example trajectory of an isolated neuron θ_*i*_ in this regime, driven only by its input *I*_*i*_(*t*). Model (1) has been analyzed previously for uncoupled neurons (Ritt, 2003; Lin et al., 2009a), and for a series of gradually more complex networks in Lin et al. (2009a,b); Lajoie et al. (2013) (cf. Monteforte and Wolf, 2010).

**Figure 2:**
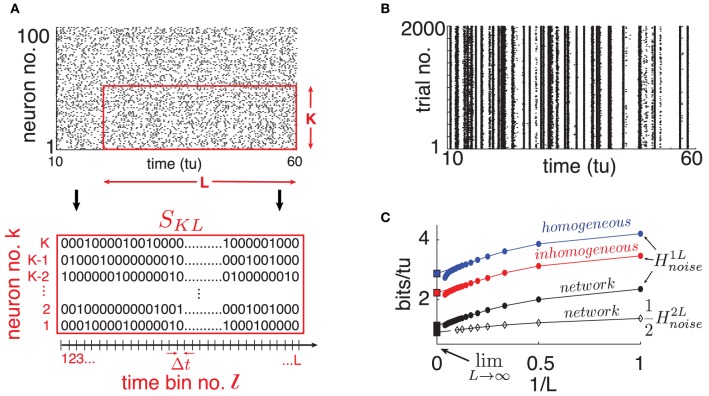
**(Color online) (A)** Top: Raster plot of spike output for 100 randomly selected neurons on a single trial (dots are spikes). Bottom: Illustration of binary *S*_*KL*_-word. **(B)** Raster plot of one randomly selected cell's spike output on 2000 trials where only network initial conditions change. **(C)** Single cell *H*^1*L*^_*noise*_ estimates for different choices of “surrogate” noise (round markers); see text. From top to bottom: homogeneous poisson (blue), inhomogeneous poisson (red), network interactions (black). The bottom curve is a computation of $\frac{1}{2}H_{noise}^{2L}$ from a cell pair (diamond markers). Abscissa scale is 1/*L* to better visualize extrapolation of extensive regime to *L* → ∞ (left square marker). For all panels: η = −0.5, ε = 0.5, *N* = 500.

We assign 20% of the *N* neurons to be inhibitory and 80% to be excitatory, meaning that outgoing weights of neuron *j* are either *a*_*ij*_ ≤ 0 or *a*_*ij*_ ≥ 0 respectively. The coupling matrix *A* = {*a*_*ij*_}_*i*,*j* = 1, …, *N*_ is chosen randomly with mean *in-degree* κ such that each neuron receives on average κ incoming connections from independently chosen neurons, from each excitatory/inhibitory population. Here, |*a*_*ij*_| ~ 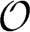
$\left(1/\sqrt{\kappa }\right)$ when non-zero, in accordance with classical *balanced state* coupling (van Vreeswijk and Sompolinsky, 1998). Throughout, we set κ = 20 (|*a*_*ij*_| ≃ 0.2) but find that as long as κ ≪ *N*, our findings are qualitatively robust to the choice of κ. Two consequences of this connectivity will be important below. First, as the mean in-degree κ is the same for all neurons, the spiking statistics of single cells are fairly stereotypical on average across the network. This is evident in the spike rasters of Figure 2A. Second, the magnitude of inputs to single cells remains similar as network size *N* grows, because κ is fixed.

We emphasize that the collection *I* is a multi-dimensional signal and not stochastic noise. We study the solutions of (1) arising from distinct initial conditions (IC) but receiving the same input *I*. In contrast to a standard stochastic differential equation, this interpretation of system (1) is defined as a *random dynamical system* (RDS) (Kunita, 1990). As we will see below, RDS theory addresses questions of ensemble dynamics when a quenched “realization” of a stochastic process drives an underlying dynamical system. This framework enables us to ask questions about stimulus-response variability of a chaotic network due to any perturbation. For example, one might ask: What is the impact of deleting or adding a spike from some neuron(s) on the future spiking output of the network (c.f. London et al., 2010)? This scenario is equivalent to comparing the response from the network initialized at a given state to its response resulting from a second perturbed state, where the coordinates of some neurons are set to be at or away from their spiking phase. Our approach is a generalization of this formulation as we consider large ensembles of initial states, and study the differences between resulting trajectories in response to a fixed input. This will enable us to quantify the statistics of variability in network responses due to chaos.

### 2.2. Spike-response noise entropy and direct estimates

To quantify spike pattern variability, we treat spike trains as binary time series. We discretize time in bins of width Δ*t* small enough so that for a given cell, each bin contains at most a single spike. Throughout, we use time bins of width Δ*t* = 0.05 time-units; we found that moderately different resolutions did not significantly affect our results. Let us define finite binary words for *K* neurons over *L* time bins starting at time *t*_*l*_ = *l*Δ*t* for some integer *l*: *S*_*KL*_(*t*_*l*_) = {*S*^*k*^_*l*_, …, *S*^*k*^_*l* + *L* − 1_}_*k* = *k*_1_, …, *k*_*K*__ with *S*^*k*^_*j*_ ∈ {0, 1} (see Figure 2A).

The variability of the evoked spike response *S*_*KL*_(*t*_*l*_) is captured by the *spike-response noise entropy*

(2) $$H_{noise}^{KL}(I,t_{l})=\frac{-1}{L\Delta t}\sum_{S_{KL}}P(S_{KL}(t_{l})|I)\log_{2}P(S_{KL}(t_{l})|I)$$

where *P*(*S*_*KL*_(*t*_*l*_)|*I*) denotes probability of observing word *S*_*KL*_(*t*_*l*_) conditioned on input *I*, given a random initial state of the network. This quantity may also be referred to as *conditional response entropy*. It is normalized to have units of bits per time-unit (*bits*/*tu*), as opposed to bits per time-bin, and thus represents an *entropy rate* in continuous time. Since the inputs *I* and network dynamics are statistically stationary processes (Lajoie et al., 2013), it follows that the expected noise entropy rate of *KL* words conditioned on any *I* from the same input distribution—controlled by the parameters η and ε—can be obtained from a long time average on any single I^*^ (see e.g., Rieke et al., 1996; Strong et al., 1998):

(3) $$H_{noise}^{KL}=\int_{I}P(I)H_{noise}^{KL}(I,t_{l})=\underset{T\to \infty }{\lim}\frac{1}{T}\sum_{l=0}^{T-1}H_{noise}^{KL}(I^{*},t_{l}).$$

As demonstrated in Strong et al. (1998) and reviewed below, (3) can be used to estimate the true entropy rate of *K*-neuron groups considered when *L* → ∞. As we will see, this is only practical for small *K*—we will need other tools to understand this quantity for entire networks (*K* = *N*). Nevertheless, we begin by applying a direct sampling approach.

To estimate the probability terms in (2), we simulate network (1) in response to a randomly chosen, quenched *I*(*t*) for 10, 000 time units and 2000 “trials,” distinguished by different ICs. Here, we wish to choose ICs from a distribution that best describes random network states, while being agnostic about its past. As discussed in Lajoie et al. (2013), we assume that system (1) possesses an ergodic stationary probability measure μ(θ), which is the steady state solution of the Fokker-Planck equation associated with (1). Thus, μ is the probability measure describing how likely we are to find the network in a particular state at any moment in time, given the history of any input *I* with identical statistics. We emphasize that μ serves only as an initial distribution, and that ensembles of “trial” trajectories as described above will have a very different distribution, as they are conditioned on a *fixed* input *I*. (See Lin et al., 2009a,b; Lajoie et al., 2013 for more details about this distinction).

To sample from μ, we first select seed ICs uniformly over the state space, and evolve each of these for a “burn” period of 50 time units, for which different inputs are presented. The resulting endpoints of these trajectories represent a new IC ensemble that approximates μ. From then on, all ICs are integrated using the same input *I* and we use this solution ensemble to study variability of spike-responses.

From these simulated network trajectories, we first discard the first 100 time-units to eliminate transient effects. We then extract the binary spike output of neurons across all trials (see Figure 2B for a single, network-embedded neuron example). Normalized cross-trial counts of *S*_*KL*_ words in consecutive, non-overlapping *L*-windows serve as estimates of the probabilities *P*(*S*_*KL*_(*t*_*l*_)|*I*) in Equation (2).

## 3. Results

### 3.1. Single-cell variability

We begin by computing noise entropy in the spike responses of single cells in the network. Using the estimation techniques described above, we compare the effect of chaos to that of commonly used independent noise models on noise entropy. This complements similar analysis in Lajoie et al. (2013), which used a different metric of spike reliability from trial to trial.

We start by randomly selecting a cell in our network and extract its binary spike output across many simulated trials (see Figure 2B). Using this data, we estimate *H*^1*L*^_*noise*_ for word lengths up to *L* = 20 and plot the results in Figure 2C as a function of 1/*L*. A system with finite autocorrelation timescales is expected to produce entropy rates that behave extensively as *L* becomes sufficiently large. This is readily apparent in the linear decreasing trend in *H*^1*L*^_*noise*_ as *L* grows, until a point where the estimate quickly drops due to insufficient sampling. Following Strong et al. (1998), we use the point of least fractional change in slope to extrapolate this extensive trend and obtain an estimate for lim_*L* → ∞_*H*^1*L*^_*noise*_ (intersection with ordinates in Figure 2C). We verified that taking larger sample sizes—with *L* up to 30 and ensembles of up to 10, 000 trials– did not significantly affect our estimates.

Our estimate of lim_*L* → ∞_*H*^1*L*^_*noise*_ is 1.12 *bits*/*tu*. We note that a “purely random,” homogeneous poisson spike train with the same firing rate (0.8 *spikes*/*tu*) would have noise entropy *H*^1*L*^_*noise*_ of 3.67 *bits*/*tu*. Thus, while chaotic dynamics produce variable spiking in single cells, the resulting noise entropy is much less than that of a totally random response, a fact also evident from the spike rasters in Figure 2B.

Part of the reason for this difference is simply the presence of the stimulus; inputs from other cells in the chaotic networks also play a role. To isolate the network effect, we repeat the sampling process above by simulating our chosen cell in isolation, keeping the input *I*_*i*_ intact but replacing the incoming spike trains it receives from upstream cells by two surrogate ensembles meant to isolate distinct statistical features of network activity. (i) *Homogeneous poisson* surrogates: independent, poisson distributed spike trains with rate matching the mean firing rate of corresponding upstream cells. (ii) *Inhomogeneous poisson* surrogates: produced by independently drawing a binary random variable in each Δ*t*-bin, according to the time-dependent probability given by the normalized spike count of the corresponding network train across all original trials. For each new simulated trial, we draw independent surrogates. Figure 2C shows a 66% increase in noise entropy rate for the homogeneous surrogates, and about 30% for the inhomogeneous case.

Overall, we have shown that single, stimulus-driven cells in chaotic networks produce spike-response entropy significantly lower than that expected for single, stimulus-driven cells receiving poisson background inputs, as in many statistical models. We next seek to characterize spike entropy in the joint responses of multiple cells.

### 3.2. Multi-cell variability

Our network is connected—albeit sparsely (κ ≪ *N*)—and it is not clear in advance how coupling interactions will impact the entropy rate of groups of cells. As a first step, we repeat the noise entropy estimate described above for a randomly selected pair of connected cells up to *L* = 10, and extrapolate lim_*L* → ∞_*H*^2*L*^_*noise*_ from this data. The black lines in Figure 2C show *H*^2*L*^_*noise*_/2, normalized to units of bits per time-unit per neuron for comparison with *H*^1*L*^_*noise*_. Due to combinatorial explosion of possible spike patterns as more neurons are considered, we were unable to compute such estimates for *K* greater than 2. Nevertheless, it appears from the *K* = 2 case shown that interactions between neurons conspire to lower response noise entropy per neuron, if only by a small margin.

However, this margin could easily be missed. For a given neuron pair (*i, j*), consider the difference between the sum of independent cell entropy rates and their joint pair rate: δ_*ij*_ = lim_*L* → ∞_[*H*^1*L*^_*noise*_(*i*) + *H*^1*L*^_*noise*_(*j*) − *H*^2*L*^_*noise*_(*i, j*)]. From 45 random pairs of neurons, we obtain the average 〈δ_*ij*_〉 = 0.012 bits/tu. This implies a relative difference of the order of 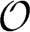
(10^−2^) when estimating the entropy rate of pairs of cells using their marginal, single-cell response distributions. We will see later these small differences compound significantly when considering the network as a whole (cf. Schneidman et al., 2006).

**Figure 3:**
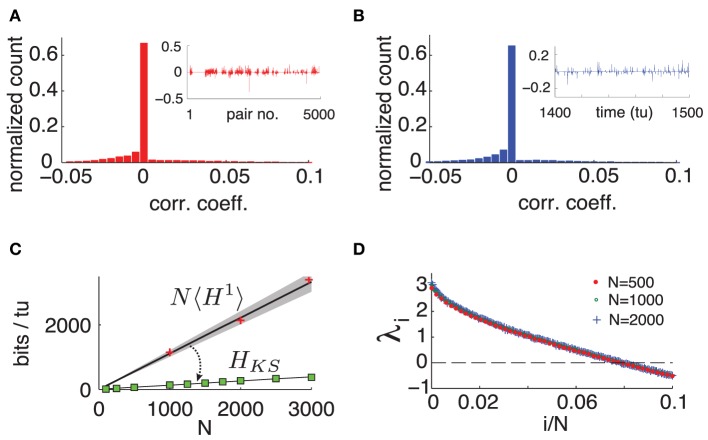
**(Color online) (A)** Typical histogram of noise correlation coefficient *c*_*ij*_(*t*_*l*_) between all neuron pairs for a fixed time. Inset shows *c*_*ij*_(*t*_*l*_) for the first 5000 pairs. **(B)** Histogram of noise correlation coefficient *c*_*ij*_(*t*_*l*_) between two connected cells across 10, 000 *tu*. Inset shows *c*_*ij*_(*t*_*l*_) for 100 tu. **(C)** Network-wide noise entropy estimates in *bits*/*tu* as a function of *N*. Slope 〈*H*^1^〉 averaged over 20 random cells in a network with *N* = 500. Shaded area shows two standard errors of the mean. Markers show direct samples from single cells for various network sizes (ie *NH*^1^). *H*_*KS*_: square markers shows estimates from Lyapunov spectra for a range of *N*; black line is a linear fit. **(D)** Plot of first 10% of Lyap spectrum for *N* = 500, 1000, and 2000. For all panels: η = −0.5, ε = 0.5.

To quantify the extent of these interactions over space and time, we compute the Pearson correlation coefficient *c*_*ij*_(*t*_*l*_) between the spiking probability of two cells *i* and *j* in time bin *t*_*l*_. That is, we measure the cells' instantaneous *noise correlation*. Figure 3A shows a typical histogram of *c*_*ij*_(*t*_*l*_) across all neuron pairs of a network with *N* = 500 for a fixed *t*_*l*_, where pairs with zero spiking probability were discarded. We can see that at a fixed moment, correlations are weak and most cells are uncorrelated. Moreover, these correlations are not static: a high correlation between two cells in one time bin does not guarantee that they will be correlated in another. This is illustrated by Figure 3B, showing a histogram of *c*_*ij*_(*t*_*l*_) across 10000 time-units between two randomly chosen connected cells.

We emphasize that this weak and highly dynamic correlation structure might easily be dismissed as negligible experimentally. If one would choose a single pair of cells and measure the temporal average of *c*_*ij*_(*t*_*l*_) over 500 time units, one obtains an average of the order of 10^−5^ (over 4950 cell pairs tested), and standard deviation of the order of 10^−2^ (across the 4950 cell pairs.) In other words, each individual cell pair appears to be almost completely uncorrelated–at least on average. Below, we will show that the weak, transient dependencies that are in fact present among neurons nevertheless have a very strong impact on network-wide noise entropy.

To summarize, measures of entropy and correlations indicate that there are noticeable but weak dependencies in the spiking activity of connected pairs of cells. Scaling up from such dependencies to accurately describe the joint activity of an entire network is a notoriously difficult problem. We take an approach based on RDS in what follows. This approach will quantify the entropy *H*^*NL*^_*noise*_ of the network as whole, as networks size *N* grows.

### 3.3. A benchmark for network entropy

To benchmark *H*^*NL*^_*noise*_ for different network sizes, we first describe the joint network entropy that would be naively predicted by direct extrapolation from single cells. In other words, this is the estimate one would obtain by ignoring statistical interactions between neurons. As the entropy of a multivariate distribution is always smaller or equal to the sum of the marginal distributions' entropies, it follows that if 〈*H*^1^〉 denotes the average of lim_*L* → ∞_*H*^1*L*^_*noise*_ over all neurons, then *N*〈*H*^1^〉 ≥ lim_*L* → ∞_*H*^*NL*^_*noise*_. We estimate 〈*H*^1^〉 by sampling lim_*L* → ∞_*H*^1*L*^_*noise*_ from randomly chosen neurons in a network with *N* = 500 using the same technique as in Figure 2C. As the mean in-degree κ for incoming connections to each neuron is constant, we found that using an ensemble of 20 neurons randomly sampled from the full network, gave a good estimate for 〈*H*^1^〉.

Unlike cell pairs, spiking statistics of single neurons are expected to be unchanged by network size *N* with fixed in-degree κ. We therefore use 〈*H*^1^〉 to extrapolate the extensive upper bound on network noise entropy *N*〈*H*^1^〉 as a function of network size *N*. Figure 3C shows this estimate, where the shaded area around the line denotes the extrapolation of two standard errors of the mean of 〈*H*^1^〉 estimated in a network with *N* = 500. We verified by spot checks that single cell activity in networks of different sizes agree with this extrapolation (see markers in Figure 3C). Next, we leverage dynamical properties of our network to estimate how much reduction in entropy can be expected from the joint activity of entire networks in comparison to this naive extensive bound.

### 3.4. Dynamical entropy production

In what follows, we use symbolic dynamics to map between the phase space of our network and the set of binary spike trains. Consider trajectories θ(*t*) = (θ_1_(*t*), …, θ_*N*_(*t*)) of model (1), evolving on the *N*-dimensional torus 𝕋^*N*^. Recall that a spike from cell *i* occurs when θ_*i*_(*t*) = 1, and will lead to *S*^*i*^_*l*_ = 1 in the corresponding time bin. Notice that the phase response curve *Z*(θ_*i*_) modulates the effect of any input on neuron *i*–whether that input comes from the signal *I*_*i*_(*t*) or from network activity—and that it vanishes at θ_*i*_(*t*) = 1. This implies that a neuron becomes insensitive to any inputs when it is about to spike. Indeed, the Taylor expansion of neuron *i*'s dynamics about θ_*i*_ = 1 is constant up to quadratic order: *d*θ_*i*_ = [2 + 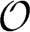
((θ_*i*_−1)^2^)]*dt* + 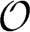
((θ_*i*_−1)^2^)*dW*_*i*,*t*_. Based on this observation we make the approximation that for Δ*t* small enough, neuron *i* spikes in the time bin [*t, t* + Δ*t*] if and only if θ_*i*_(*t*) ∈ [1 − 2Δ*t*, 1) (see next section for verification).

Thus, equipped, consider the following partition of the state space 𝕋^*N*^: Γ^*^ = {γ_0_, γ_1_}^*N*^, built of Cartesian products of intervals γ_0_ = [0, 1 − 2Δ*t*) and γ_1_ = [1 − 2Δ*t*, 1) across all θ_*i*_'s. At any time *t*_*l*_ = lΔ*t*, the Γ^*^-address of θ(*t*_*l*_) determines the binarized spiking state of the network in time bin [*t*_*l*_, *t*_*l*_ + Δ*t*]: θ_*i*_(*t*_*l*_) ∈ γ_0_ ⇒ *S*^*i*^_*l*_ = 0 and θ_*i*_(*t*_*l*_) ∈ γ_1_ ⇒ *S*^*i*^_*l*_ = 1. In order to describe *L*-long spike trains in terms of Γ^*^-addresses, we must understand how solutions θ(*t*) evolve with respect to Γ^*^. To this end, consider the discretized dynamics given by the transition maps Φ_*t*;*I*_ that send 𝕋^*N*^ onto itself according to the flow of (1) from *t* to *t* + Δ*t*. If θ(*t*) is a solution of (1), then Φ_*t*;*I*_(θ(*t*)) = θ(*t* + Δ*t*) where Δ*t* refers to the resolution of our binary spike trains *S*_*NL*_. Note that the maps Φ_*t*;*I*_ depend on both *t* and *I*, are generally smooth with smooth inverses (diffeomorphisms) (Kunita, 1990), and together form a discrete RDS. For detailed geometric properties of the RDS defined by system (1), we refer the reader to Lajoie et al. (2013).

For what follows, it is convenient to reverse time and study spike trains and trajectories starting in the distant past leading up to *t* = 0. This representation is statistically equivalent to forward time since our network has stationary dynamics (Lajoie et al., 2013). Consider now the *l*-step inverse map: Φ^−*l*^_0;*I*_. For any set *A* in the partition Γ^*^, its pre-image Φ^−*l*^_0;*I*_(*A*) refers to all points in 𝕋^*N*^ at time −*l*Δ*t* that will be mapped to *A*, and consequently have the same spiking state at *t* = 0. Similarly, if both *A*_0_ and *A*_1_ are sets in Γ^*^, the intersection Φ^−*l*^_0;*I*_(*A*_0_) ∩ Φ^−*l*+1^_0;*I*_(*A*_1_) describes all points that will be mapped to *A*_1_ at *t* = −Δ*t* and *A*_0_ at *t* = 0. It follows that any subset of the form *B* = ∩^*L*^_*l* = 0_ Φ^−*l*^_0;*I*_(*A*_*l*_) where *A*_*l*_ ∈ Γ^*^ captures all past network states at time *t* = (−*L*)Δ*t* leading to identical spiking sequences {*S*^*i*^_−*L*_, …, *S*^*i*^_−1_, *S*^*i*^_0_}_*i* = 1, …, *N*_, when the same *I* is presented. Moreover, it is easy to show that the collections of all possible sets constructed as *B*, named the *join* of pre-images of Γ^*^ and denoted ∨^*L*^_*l* = 0_Φ^−*l*^_0;*I*_Γ^*^, is itself a partition of 𝕋^*N*^.

It follows that this new partition offers a one-to-one correspondence between its member sets and the space of all *S*_*NL*_ spike trains. Note that many sets in this partition will be empty since not all spike sequences are accessible by the network. In fact, the number of non-empty sets remaining in ∨^*L* − 1^_*l* = 0_Φ^−*l*^_0;*I*_Γ^*^ as *L* → ∞ represents the number of allowed infinite spike sequences. Furthermore, for a given *S*_*NL*_ and its associated set *B*(*S*_*NL*_) ∈ ∨^*L* − 1^_*l* = 0_Φ^−*l*^_0;*I*_Γ^*^, the probability of observing spike pattern *S*_*NL*_ can be stated as an initial state probability in the distant past: *P*(*S*_*NL*_|*I*) = *P*(θ(−*L*Δ*t*) ∈ B(*S*_*NL*_)).

As discussed above and in Lajoie et al. (2013), we assume that our RDS possesses an ergodic stationary probability measure μ. Recall that we assume random ICs forming our distinct trials are drawn from μ. It follows that lim_*L* → ∞_ *P*(*S*_*NL*_|*I*) = μ(*B*(*S*_*NL*_)). Thus, if we let

(4) $$h_{\mu }(\Phi_{t;I},\Gamma^{*})=\underset{L\to \infty }{\lim}-\frac{1}{L}\sum_{B\in \vee_{l=0}^{L}\Phi_{0;I}^{-l}\Gamma^{*}}\mu (B)\ln\mu (B),$$

it follows that

(5) $$\underset{L\to \infty }{\lim}H_{noise}^{NL}=\frac{\Delta t}{\ln2}h_{\mu }(\Phi_{t;I},\Gamma^{*}).$$

For any dynamical system, the expression (4) measures the amount of uncertainty produced by chaotic dynamics if we can only observe the system with the precision given by the partition Γ^*^. This concept is generalized by the *Kolmogorov-Sinai entropy h*_μ_, also called *dynamical* or *metric* entropy (Ruelle, 1989; Greven et al., 2003), defined by

(6) $$h_{\mu }=\underset{\Gamma }{\sup}h_{\mu }(\Phi_{t;I},\Gamma )$$

where the supremum is taken over all finite partitions Γ. This quantity is related to the Lyapunov spectrum λ_1_ ≥ λ_2_ ≥ ··· ≥ λ_*N*_ of a dynamical system which measures rates of exponential divergence or convergence between trajectories. Lyapunov exponents λ_*i*_ are expected to be well defined for our RDS in the sense that they rely on system parameters such as coupling strength and the mean and variance of inputs, but not on specific realizations of the inputs *I*(*t*) (Kifer, 1986). The authors of Ledrappier and Young (1988) showed that although the join of a partition Γ depends on *I*, *h*_μ_ does not and that under some ergodicity assumptions, the following entropy formula holds:

(7) $$h_{\mu }=\sum_{\lambda_{i}>0}\lambda_{i}.$$

If λ_*i*_ are the Lyapunov exponents of the original system (1) computed over time-units instead of Δ*t* time-steps, we get from (4), (5), (6), and (7) the following upper bound for noise entropy rate:

(8) $$H_{KS}\equiv \frac{1}{\ln2}\sum_{\lambda_{i}>0}\lambda_{i}\geq \underset{L\to \infty }{\lim}H_{noise}^{NL}$$

which has units of bits per time-unit.

To evaluate this bound, we numerically compute the exponents λ_*i*_ of system (1) and find that, as originally observed in Monteforte and Wolf (2010) and Luccioli et al. (2012) for autonomous networks, our driven system has a size invariant Lyapunov spectrum (see Figure 3D), which is insensitive to particular choices of random coupling matrix *A* (see Supplementary Material for details). This leads to a spatially extensive behavior of the bound *H*_*KS*_, as shown in Figure 3C.

Intriguingly, *H*_*KS*_ is much smaller than estimates from 〈*H*^1^〉. This reveals a central result for our driven chaotic networks: *joint spike patterns are (at least) an order of magnitude less variable than what would be predicted by observing the spike train statistics of single cells, despite averaged noise correlations across neurons that are very low*.

### 3.5. Relationship between state space partitioning and spiking patterns

The derivation of the *H*_*KS*_ bound (8) relies on the simple assumption that neuron *i* will spike within Δ*t* time-units if and only if θ_*i*_(*t*) ∈ γ_1_ = [1 − 2Δ*t*, 1]. As discussed above, this assumption holds in the limit of small Δ*t*. We found that for simulated trajectories of 1000 time-units from network (1), only about 0.01% of all spikes violated the spiking assumption for Δ*t* = 0.05. This number dropped to zero for Δ*t* = 0.01. Such values are evidence that errors in relating spike train entropy estimates to entropy production in state space will be slight. In the present section, we verify this in detail.

To do so, we compare the spiking statistics and entropy estimates for the main model (1) with those for an analogous dynamical system, for which our partition-based spiking assumption holds exactly, by design. Consider the *piecewise* model analogous to system (1):

(9) $$\begin{array}{l} d\theta_{i}=[\tilde{F}(\theta_{i})+\tilde{Z}(\theta_{i})\sum_{j=1}^{N}a_{ij}g(\theta_{j})+\frac{\varepsilon^{2}}{2}\tilde{Z}(\theta_{i}){\tilde{Z}}^{\prime }(\theta_{i})]dt\ldots \\ \;+\;\tilde{Z}(\theta_{i})\underset{I_{i}(t)}{\underset{︸}{\left[\eta dt+\varepsilon dW_{i,t}\right]}} \end{array}$$

in which we replace the functions *F* and *Z* by the following piecewise-defined terms:

$$\begin{array}{l} \tilde{F}(\theta_{i})=\left\{ \begin{array}{l} 1+\cos(2\pi \theta_{i})\text{ ; }\theta_{i}\in [0,1-2\Delta t)\\ \;2\text{ ; }\theta_{i}\in [1-2\Delta t,1) \end{array}\right.\\ \tilde{Z}(\theta_{i})=\left\{ \begin{array}{l} 1-\cos(2\pi \theta_{i})\text{ ; }\theta_{i}\in [0,1-2\Delta t)\\ \;0\text{ ; }\theta_{i}\in [1-2\Delta t,1). \end{array}\right. \end{array}$$

It is easy to see that the partition-based spiking assumption holds exactly for the network defined by (9). However, notice that for Δ*t* > 0, both $\tilde{F}$ and $\tilde{Z}$ are discontinuous functions of *S*^1^ and that as a result, the Jacobian of (9) is ill-defined. Nevertheless, for practical purposes, we can simulate system (9) and approximate its Lyapunov spectrum, since there is only one discontinuity point per neuron and the probability of a finite-duration, discretized trajectory landing on such points is nil.

**Figure 4:**
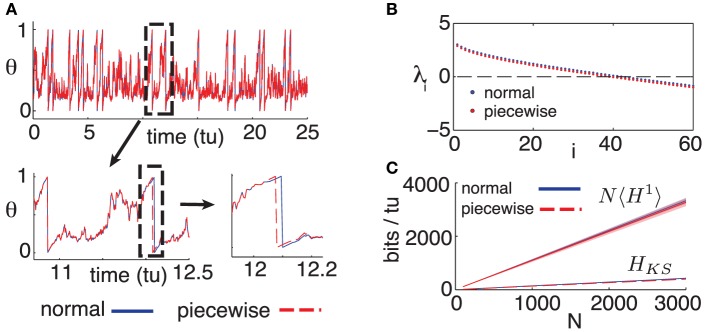
**(A)** Comparison of trajectories for single cells, for models (1) and (9); initial conditions and inputs are fixed. **(B)** First 60 Lyapunov exponents of models (1) and (9). **(C)** Empirical noise entropy bounds *NH*^1^ and *H*_*KS*_ for models (1) and (9). For all panels, η = −0.5, ε = 0.5, Δ*t* = 0.05. For **(B,C)**, *N* = 500, κ = 20.

The purpose of model (9) is to assess the differences arising between the dynamics of our full (“normal”) model, given by Equation (1), and the alternate (“piecewise”) model above for which the spiking assumption is exact. We fix Δ*t* = 0.05 as in the main text and begin by comparing single cell dynamics for the “normal” and “piecewise” models. Figure 4 shows a simulated single cell trajectory from each model, with identical input *I*_*i*_ and identical incoming spike trains (extracted from a separate network simulation). This setup mimics the activity a single cell would receive when embedded in a network. Notice that apart from small discrepancies that sometimes arise between spike times, the two trajectories agree almost perfectly. When differences do arise, they are quite small. From a simulation yielding about 3000 spikes from both models, most corresponding spikes from the normal and piecewise models were indistinguishably close, down to the numerical solver's time-step. The maximal difference was about 0.02 time-units, smaller than a Δ*t* time-bin.

Figure 4B shows the first 60 Lyapunov exponents of a network with size *N* = 500, simulated with both the normal (1) and piecewise (9) models. Since Lyapunov exponents depend on the Jacobian of a system, we expected the piecewise model to yield smaller exponents: its derivative is zero on the intervals [1 − 2Δ*t*, 1). Nevertheless, this discrepancy is minimal and amounts to a difference of about 0.002 bits per neuron per time-unit in the slope of the *H*_*KS*_ estimates shown in Figure 4C. Finally, we empirically estimate the noise entropy bound 〈*H*^1^〉, as described in the main text, for the piecewise model (9). Its value differed from the normal model estimate by about 0.01 bits per neuron per time-unit, well below the standard error of the mean of estimates from both models, as can be seen in Figure 4C.

In light of these tests, we are confident that the main result of the paper—a computable bound on spike-train noise entropy that is much lower than what would be extrapolated from single cells—is a robust phenomenon for networks of the type modeled by (1), rather than a consequence of a (seemingly tiny) approximation error.

### 3.6. Noise entropy production as a function of input statistics

**Figure 5:**
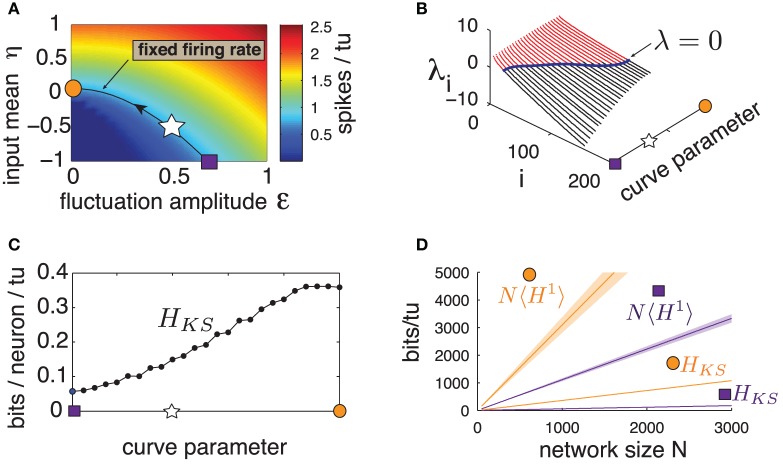
**(Color online) (A)** Heat map of excitatory population mean firing rate for a range of input amplitude ε and input mean η. Line is the contour curve for fixed firing rate of 0.820 spikes/tu ± 0.003, parameterized by numerical interpolation. Arrow shows direction of parametrization. Markers: square: η = −1, ε = 0.69, star: η = −0.5, ε = 0.5, circle: η = 0.07, ε = 0. **(B)** Lyapunov spectra along contour curve from **(A)**. **(C)** *H*_*KS*_ bounds evaluated along contour curve from **(A)**. **(D)** Network noise entropy bounds *N*〈*H*^1^〉 and *H*_*KS*_ for square and circle marker parameters in **(A)**. Slope 〈*H*^1^〉 averaged over 20 random cells. Shaded area shows two standard errors of the mean. Both 〈*H*^1^〉 and *H*_*KS*_ extrapolated from a network with *N* = 500, as are quantities from all other panels.

Previous studies showed that the level of sensitivity emerging from chaotic network dynamics can be controlled by carefully chosen inputs (see Molgedey et al., 1992; Rajan et al., 2010 for different contexts). We verify if this is the case for our network. We first identify a range of input statistics—the mean η and fluctuation amplitude ε—that are comparable in that they all produce the same firing rate as for the “standard” parameter set used above (η = −0.5, ε = 0.5). These parameters lie along the level curve in Figure 5A. Note that the curve is parameterized so that η grows while ε decreases; thus, as we travel along it, we gradually shift the dynamics from the excitable, fluctuation-driven regime (η < 0) to an oscillatory, mean-driven one (η > 0). In particular, the last point evaluated corresponds to a purely autonomous regime (ε = 0) where the input *I* has no fluctuating component.

Figure 5B shows the first 200 Lyapunov exponents of a network with *N* = 500 along this level curve, and panel (c) gives the corresponding *H*_*KS*_ values. A clear trend emerges: *H*_*KS*_ increases monotonically as the system transitions from fluctuation- to mean-driven regimes, by almost an order of magnitude. Moreover, Figure 5D shows that, for the two extremes of the level curve, network noise entropy continues to be much smaller than that predicted from single cells, and that single-cell noise entropy appears to follow the same trends as *H*_*KS*_. We conclude that *spike pattern variability emerging from chaos is not a fixed property of a network, but can be strongly modulated by the mean and variance of network inputs*.

## 4. Discussion

Biological neural networks may operate in a chaotic regime, with irregular activity driven by a balance of fluctuating excitatory and inhibitory interactions. This network chaos is under vigorous study, fueled in part by possible roles for chaos in generating “target” spatiotemporal patterns (Sussillo and Abbott, 2009) and in enabling useful temporal processing of inputs (Buonomano and Maass, 2009; Laje and Buonomano, 2013). Here, we address a complementary question: How much variability (or “noise”) will chaotic dynamics add to network responses?

We compute bounds on network spike-response entropy that give novel answers. In particular, we show that the noise entropy of multi-cell spike responses is at least an order of magnitude lower than would be naively extrapolated from from single-cell measurements, under the assumption that spike variability is independent from cell to cell. The direction of the comparison between noise entropy of single cell and multi-cell spike responses agrees with intuition provided by the shape of the Lyapunov spectrum, which indicates time-dependent chaotic attractors of lower dimension than phase space. Thus, the phase space dynamics of each neuron are not independent. What we quantify explicitly is the order-of-magnitude size of the effect, as it is manifested in the binary *spiking* outputs of the system—a fact which might seem especially striking given that pairs of spike trains appear to be very weakly correlated on average.

If one considers the level of noise entropy as an indicator of potential information contained in spike patterns, we show that balanced networks may be able to encode inputs stimuli using spike timing if these inputs contain strong enough temporal structure. This mechanism takes root in the complex noise-interactions that chaos induces between neurons. The extensive nature of this phenomenon suggests that this mechanism is scalable with network size. Moreover, the strong dependence of entropy on the input signal's mean and variance indicate that a network can operate in different “regimes” modulating the repeatability of spike patterns. This is in addition to known advantages of balanced networks, such as efficiently tracking changes in common, mean inputs with firing rates (van Vreeswijk and Sompolinsky, 1998)—which may encode coarser statistics about inputs at the population level.

To formalize these notions, future work could seek to compute the mutual information between an input ensemble and a system's response. In order to estimate this quantity, one needs to compute the *total entropy* (Rieke et al., 1996) of spike patterns—in addition to the noise entropy computed in this paper– which captures how many distinct spike outputs can be produced by the network, for any input *I*. This quantity can be thought of as noise entropy marginalized over the set of possible inputs. Estimating the total entropy in large networks is a difficult problem since it depends on the evolution of ensembles of trajectories driven by ensembles of inputs. In other words, one needs to capture the entropy of trajectories when system (1) is treated as a stochastic differential equation rather than a RDS, a distinction that introduces a variety of challenges.

Our results complement prior work on the behavior of sparse, balanced networks in the large *N* limit. Seminal results use mean-field approaches (e.g., van Vreeswijk and Sompolinsky, 1998), deriving successful estimates of population activity statistics such as the mean and the variance of firing rates. In this approach, self consistent equations are derived for representative single cells based on the assumption that, when *N* is sufficiently large and *k*/*N* is sufficiently small, the inputs to each neuron in the network can be approximated by independent gaussian noise. In contrast, we derive estimates for the impact of correlations among these individual cells. Interestingly, in both the classical and the present work, noise entropy scales extensively with *N*; here, the predicted rate of scaling would be lower, as even weak correlations between cells combine to create statistical dependencies—especially when network activity is conditioned on an input.

Finally, we expect that the *H*_*KS*_ bound can be adapted to other neuron models, provided a state space partition linking dynamics to spike patterns can be derived. This could prove to be a powerful tool to investigate stimulus encoding as a function of many network attributes, such as spike-generating dynamics, connectivity, learning rules and input correlations.

## Funding

This work was supported in part by an NIH Training Grant from University of Washington's Center for Computational Neuroscience (5R90DA03346103, 5T90DA03243602), a Bernstein Fellowship from the Bernstein Center for Computational Neuroscience, a postdoctoral scholarship from the *Fonds de Recherche du Québec*, the Burroughs Wellcome Fund for Scientific Interfaces, the NSF under grant DMS CAREER-1056125 and NSERC Discovery and CIHR operating grants. Numerical simulations were performed on NSF's XSEDE supercomputing platform.

### Conflict of interest statement

The authors declare that the research was conducted in the absence of any commercial or financial relationships that could be construed as a potential conflict of interest.
