# Supplementary material for: Structured chaos shapes spike-response noise entropy in balanced neural networks
Source: Front Comput Neurosci. 2014 Oct 2;8:123. doi: 10.3389/fncom.2014.00123 (PMC4183092; doi:10.3389/fncom.2014.00123)
Supplement: Supplementary file 1 [file Presentation1.PDF]

Supplementary Material for: Structured chaos shapes spike-response noise entropy in balanced neural networks

Guillaume Lajoie
Jean-Philippe Thivierge
Eric Shea-Brown

Numerical simulations

Throughout the main text, we use data from numerical simulations of the network model described by

$$\begin{aligned} d\theta_i =& [F(\theta_i) + Z(\theta_i) \sum_{j=1}^{N} a_{ij} g(\theta_j) + \frac{\varepsilon^2}{2} Z(\theta_i) Z'(\theta_i)] dt ... \\ &+ Z(\theta_i) \underbrace{[\eta dt + \varepsilon dW_{i,t}]}_{I_i(t)dt} \end{aligned} \tag{1}$$

where $F(\theta_i) = 1 + \cos(2\pi\theta_i)$, $Z(\theta_i) = 1 - \cos(2\pi\theta_i)$ and

$$g(\theta_j) = \begin{cases} d\left(b^2 - \left[\left(\theta_i + \frac{1}{2}\right) \bmod 1 - \frac{1}{2}\right]^2\right)^3 & ; \theta_i \in [-b, b] \\ 0 & ; \text{else.} \end{cases}$$

All simulations were implemented using a standard Euleur-Maruyama solver with time-steps of 0.005 time-units. We found that using smaller time-steps did not alter our results. The solver was developed using the Python/Cython programming language using the Mersenne Twister random number generator and post-processing (spike binning and empirical noise entropy estimates) was carried out in MATLAB. Large simulations were performed on the NSF XSEDE *Science Gateways* supercomputing platform.

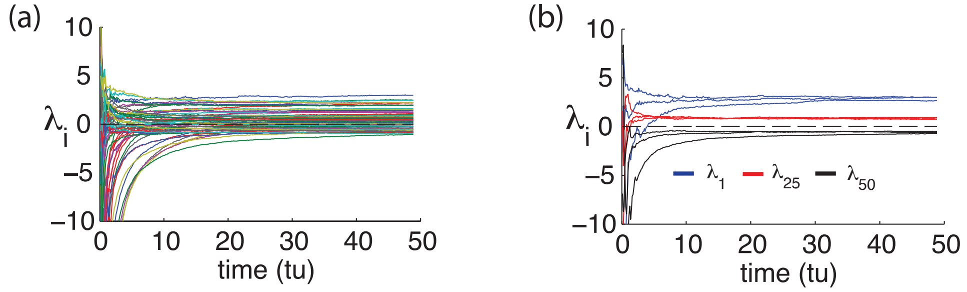

Figure 1: Estimates of Lyapunov exponents for the initial 50 out of 5000 time-units, showing convergence. (a) Estimates of the first 60 Lyapunov exponents (out of 500) for a given network. (b) Three distinct estimates for λ_1, λ_{25} and λ_{50} where network IC, I and coupling matrix A are selected differently and at random. For both panels, $N = 500$, $\varepsilon = 0.5$, $\eta = -0.5$.

Lyapunov spectrum estimates

Although the Lyapunov exponents $\lambda_1 \geq \lambda_2 \geq ... \geq \lambda_N$ of (1) do not depend on a particular choice of I or initial conditions (IC), computing them analytically is a very hard, if not an impossible, problem. Therefore, we use numerical estimates. While numerically integrating a solution of (1) above, we simultaneously evolve the linear variational equation

$$\dot{M} = J(t)M \tag{2}$$

where $J(t)$ is the Jacobian of (1) evaluated along the simulated trajectory. Here, M is a N by N matrix where $M(0)$ is the identity. $M(t)$ is orthonormalized at each time-step and the growth factors of each orthogonal vector obtained from the process are extracted to build estimates that converge toward the λ_i's, as described in [2]. This process was repeated for ten random choices of the input I and the initial states; trajectories were integrated for 5000 time-units. We verified that all reported λ_i's have a standard error less than 0.002 using the method of batched means [1] (batch size of 100 time-units). Figure 1 (a) shows converging estimates of the first 60 Lyapunov exponents over the initial 50 time-units.

In addition, we find that distinct realizations of connectivity matrix $A = \{a_{ij}\}$ did not significantly affect the Lyapunov exponent estimates — and

hence the sum of all positive ones leading to the Kolmogorov-Sinai entropy h_μ. To illustrate this, Figure 1 (b) shows estimates of three λ_i's for three distinct systems, where input choice I, IC and A are all different.

References

[1] S Asmussen and P W Glynn. *Stochastic simulation : algorithms and analysis*, volume 57 of *Stochastic modelling and applied probability*. New York: Springer, 2007.

[2] K Geist, U Parlitz, and W Lauterborn. Comparison of different methods for computing lyapunov exponents. *Prog. Theor. Phys.*, 83(5):875–893, 1990.
